# The Role of Glycoprotein 130 Family of Cytokines in Fetal Rat Lung Development

**DOI:** 10.1371/journal.pone.0067607

**Published:** 2013-06-24

**Authors:** Cristina Nogueira-Silva, Paulina Piairo, Emanuel Carvalho-Dias, Carla Veiga, Rute S. Moura, Jorge Correia-Pinto

**Affiliations:** 1 Life and Health Sciences Research Institute, School of Health Sciences, University of Minho, Braga, Portugal; 2 Life and Health Sciences Research Institute/3B's - PT Government Associate Laboratory, Braga/Guimarães, Portugal; 3 Department of Obstetrics and Gynecology, Hospital de Braga, Braga, Portugal; 4 Department of Urology, Hospital de Braga, Braga, Portugal; 5 Department of Pediatric Surgery, Hospital de Braga, Braga, Portugal; Children's Hospital Los Angeles, United States of America

## Abstract

The glycoprotein 130 (gp130) dependent family of cytokines comprises interleukin-6 (IL-6), IL-11, leukemia inhibitory factor (LIF), cardiotrophin-like cytokine (CLC), ciliary neurotrophic factor (CNTF), cardiotrophin-1 (CT-1) and oncostatin M (OSM). These cytokines share the membrane gp130 as a common signal transducer. Recently, it was demonstrated that IL-6 promotes, whereas LIF inhibits fetal lung branching. Thus, in this study, the effects on fetal lung morphogenesis of the other classical members of the gp130-type cytokines (IL-11, CLC, CNTF, CT-1 and OSM) were investigated. We also provide the first description of these cytokines and their common gp130 receptor protein expression patterns during rat lung development. Fetal rat lung explants were cultured *in vitro* with increasing concentrations of IL-11, CLC, CNTF, CT-1 and OSM. Treated lung explants were morphometrically analyzed and assessed for MAPK, PI3K/AKT and STAT3 signaling modifications. IL-11, which similarly to IL-6 acts through a gp130 homodimer receptor, significantly stimulated lung growth via p38 phosphorylation. On the other hand, CLC, CNTF, CT-1 and OSM, whose receptors are gp130 heterodimers, inhibited lung growth acting in different signal-transducing pathways. Thus, the present study demonstrated that although cytokines of the gp130 family share a common signal transducer, there are specific biological activities for each cytokine on lung development. Indeed, cytokine signaling through gp130 homodimers stimulate, whereas cytokine signaling through gp130 heterodimers inhibit lung branching.

## Introduction

Normal lung development is particularly dependent on tightly regulated signaling networks, triggered by both its classically known effectors, such as growth factors, extracellular matrix molecules and hormones, and by its recently implicated regulatory factors like inflammatory cytokines [1]–[4].

The glycoprotein 130 (gp130) dependent family of cytokines or interleukin 6 (IL-6) family of cytokines is quite a large group of structurally related cytokines that includes IL-6, IL-11, leukemia inhibitory factor (LIF), ciliary neurotrophic factor (CNTF), cardiotrophin-1 (CT-1), cardiotrophin-like cytokine (CLC), and oncostatin M (OSM) [5], [6]. Other family members have recently emerged (IL-27 and neuropoietin), thus it is likely that the currently defined gp130 cytokine family is not complete [5], [7], [8]. These small proteins are grouped in same family, since all signal through a common signal transducing receptor chain, the gp130. However, each cytokine interacts with a specific receptor that is a complex of receptor subunits. Thus, the multimeric receptor complex for gp130 family of cytokines consists of (i) gp130 homodimers with a ligand-specific α chain for IL-6 and IL-11; (ii) gp130 heterodimers (gp130/LIFR and gp130/OSMR) without specific α chain for LIF and OSM; or (iii) gp130 heterodimers with a ligand-specific α chain (CNTFRα) for CNTF and CLC or α chain-like for CT-1 [5]–[7]. Until the moment, the α chain recruited by CT-1 has not been characterized [5].

The gp130 cytokine receptors signal directly through the Janus kinase-signal transducer and activator of transcription (JAK-STAT) pathway, particularly STAT3 and STAT1 [6], [9]. Alternatively, gp130 cytokine family can also initiate cell signaling via other signaling pathways, including the mitogen-activated protein kinase (MAPK) and phosphatidylinositol-3 kinase (PI3K/AKT) cascades [6], [10], [11]. Through these pathways, gp130 cytokine signaling activates target genes involved in several cellular responses namely, cell differentiation, survival, apoptosis and proliferation. Concomitant with these responses, negative regulation of cytokine function is critical to prevent the deleterious biological consequences of excessive stimulation, and the suppressor of cytokine signaling proteins (SOCS) are well-recognized for contributing significantly to this process [6], [12]–[14]. In particular, inhibitors such as SOCS3 regulate cytokine-induced STAT3 activation by a classical negative feedback loop.

Adding to their reputation as classical regulators of immune response and inflammation, these cytokines are also well known for their regulatory role in diverse biological processes including, hematopoiesis, mammalian fertility, liver and neuronal regeneration, myocardial development, pituitary proliferation, bone homeostasis, adipocyte differentiation and function, and embryonic development [5], [6], [10], [11], [15]. Concerning lung development, IL-6 was demonstrated to have an enhancing effect on lung explant growth and proved to be an important regulator of normal lung growth, whereas in opposition to IL-6, LIF was found to inhibit lung branching [3], [4], [16]. Such evidences lead us to speculate that other members of gp130 family of cytokines might be involved in normal lung development. Moreover, these cytokines present some functional redundancy, even though they also exhibit specific biological activities [6], [11]. Therefore, we proposed to investigate the role of other gp130 family of cytokines on fetal lung growth.

## Materials and Methods

This study was carried out in strict accordance with the recommendations in the ‘Guide for the Care and Use of Laboratory Animals’, published by the US National Institutes of Health (NIH Publication No. 85–23, revised 1996). Animal experiments were also performed according to the Portuguese law for animal welfare and the protocol was approved by the Committee on the Ethics of Animal Experiments of the Life and Health Sciences Research Institute of the University of Minho (DGV 022162 - 520/000/000/2006). Moreover, all efforts were made to minimize animal suffering.

### Animal model and experimental design

Sprague-Dawley female rats (225 g; Charles-River, Spain) were maintained in appropriate cages under temperature-controlled room (22–23°C) on 12 hours light: 12 hours dark cycle, and fed with commercial solid food. The rats were mated and checked daily for vaginal plug. The day of plugging was defined as gestational day 0.5 for time dating purposes. Fetuses were removed by caesarean section at 13.5 dpc (days post-conception), sacrificed by decapitation and their lungs dissected for fetal lung explant cultures.

### Immunohistochemistry

Immunostaining was performed on paraformaldehyde-fixed and paraffin-embedded excised lungs and embryos of different gestational ages (13.5–21.5dpc). Five µm sections were placed onto glass microscope slides. Primary antibodies for IL-11 [1∶200; IL-11 (H-169), Santa Cruz Biotechnology Inc., USA], CLC [1∶50; NNT-1/BSF-3 (FL-225), Santa Cruz Biotechnology Inc.], CNTF [1∶200; CNTF (R-20), Santa Cruz Biotechnology Inc.], CT-1 [4 µg/mL; CT-1 (3G6D9), Abcam Inc., UK], OSM [1∶25; OSM (A-9), Santa Cruz Biotechnology Inc.] and gp130 receptor [1∶100; GP130 (H-255), Santa Cruz Biotechnology Inc.] were used. Tissue sections were deparaffinized in xylene and rehydrated in ethanol, boiled in 10 mM citrate buffer for antigen retrieval and cooled down at room temperature. Incubation with the primary antibody occurred at 4°C overnight. Negative control reactions included omission of the primary antibody and the simultaneous omission of the primary and secondary antibodies, in both cases immunoreactive cytokine staining was not observed. Sections were incubated with a labeled streptavidin-biotin immunoenzimatic antigen detection system (UltraVision Large Volume Detection System Anti-Polyvalent, Horseradish Peroxidase, Lab Vision Corporation, USA) according to manufacturer's instructions. For visualization of the immune reaction, a diaminobenzidine tetrahydrochloride solution (Dako, Denmark) was used. Sections were finally counterstained with hematoxylin. The slides were observed and photographed with Olympus BX61 microscope (Olympus, Japan). At least three independent experiments were performed, in each a different set of slides comprising the whole range of gestational ages plus adult, obtained from different individual samples, was used.

### Fetal lung explant cultures

Harvesting and dissection of 13.5 dpc lungs was made in DPBS (Lonza, Switzerland) under a dissection microscope (Leica MZFLIII, Switzerland). The lungs were transferred to Nucleopore membranes with an 8 µm pore size (Whatman, USA), previously presoaked in DMEM (Invitrogen, UK) for 1 hour, and incubated in a 24-well culture plates (Nunc, Denmark). Floating cultures of the explants were incubated in 200 µL of 50% DMEM, 50% nutrient mixture F-12 (Gibco, USA) supplemented with 100 µg/mL streptomycin, 100 units/mL penicillin (Gibco), 0.25 mg/mL ascorbic acid (Sigma-Aldrich, USA) and 10% FCS (Gibco). The fetal lung explants were incubated in a 5% CO_2_ incubator at 37°C for 96 hours, and the medium was replaced every 48 hours. The branching morphogenesis was monitored daily by photographing the explants. At day 0 (D_0_: 0 hours) and day 4 (D_4_: 96 hours) of culture, the total number of peripheral airway buds (branching) in all lung explants was determined, by counting the number of peripheral airway epithelial buds of the developing respiratory tree. Three additional morphometric parameters were assessed using AxionVision Rel. 4.3 (Carl Zeiss, Germany) imaging software: (1) epithelial perimeter which relates to the contour of the internal airways of the explant and is defined by the folded epithelial surface, (2) explant area and (3) external perimeter both defined by the outer edge of the whole fetal lung explant. These results were expressed as D_4_/D_0_ ratio.

### IL-11, CLC, CNTF, CT-1 and OSM supplementation studies

*In vitro* cultures were daily supplemented with several doses of recombinant IL-11 (0.1; 1; 10; 100 pg/mL), CLC (0.003; 0.03; 0.3; 3; 30 nM), CNTF (0.1; 1; 10; 100; 1000 ng/mL), CT-1 (0.1; 1; 10; 100; 200 ng/mL), and OSM (0.1; 1; 10; 100 ng/mL). All recombinant proteins were purchased from R&D Systems (USA). Per each tested dose at least nine, often more, fetal lung explants were used; likewise twelve lung explants were used as control. For CLC and CT-1 supplementation studies the control explants were supplemented with 4 mM of sterile HCl (according to manufacturer's instructions of recombinant proteins reconstitution). Sampled lung explants were obtained in three independent experiments performed.

After 4 days in culture, control and cytokine treated lung explants at selected concentrations (IL-11 at 0.1 pg/mL; CLC at 30 nM; CNTF at 1000 ng/mL; CT-1 at 200 ng/mL; OSM at 100 ng/mL) were processed for western blot analysis according to the method described below.

### Western blot analysis

Pooled samples of the cultured lung explants were processed for western blot analysis. Proteins were obtained according to Kling *et al* [17]. Ten µg of protein were loaded onto 10% acrylamide minigels, electrophoresed at 100 V at room temperature and then transferred to nitrocellulose membranes (HybondTM -C Extra, GE Healthcare Life Sciences, UK). Blots were probed with antibodies to non-phosphorylated and phosphorylated forms of p38, p44/42 (ERK1/2), JNK, AKT and STAT3 (1∶1000; Cell Signaling Technology Inc.), additionally SOCS3 (1 µg/mL; Abcam Inc.), PCNA [Proliferating cell nuclear antigen] (1∶500; Santa Cruz Biotechnology Inc.) and PARP [Poly (ADP-ribose) polymerase] (1∶1000; BD Biosciences, USA) antibodies were also used according to the manufacturer's instructions. For loading control, blots were probed with β-tubulin (1∶150000, Abcam). Afterwards blots were incubated with a secondary horseradish peroxidase conjugate (Santa Cruz Biotechnology Inc.), developed with Super Signal West Femto Substrate (Pierce Biotechnology, USA) and the chemiluminescent signal was captured using the Chemidoc XRS (Bio-Rad, USA). Quantitative analysis was performed with Quantity One 4.6.5 1-D Analysis Software (Bio-Rad). Three independent experiments were performed (n = 3).

### Statistical analysis

All quantitative data are presented as mean ± SEM. Statistical analysis was performed using the statistical software SigmaStat (version 3.5; Systat Software Inc., USA). For supplementation studies one-way ANOVA was used and for intracellular signaling pathways analysis *t-test* was used. The Student-Newman-Keuls test was used for post-test analysis. Statistical significance was set at p<0.05.

## Results

### Gp130 cytokines expression pattern during rat fetal lung development

**Figure 1:**
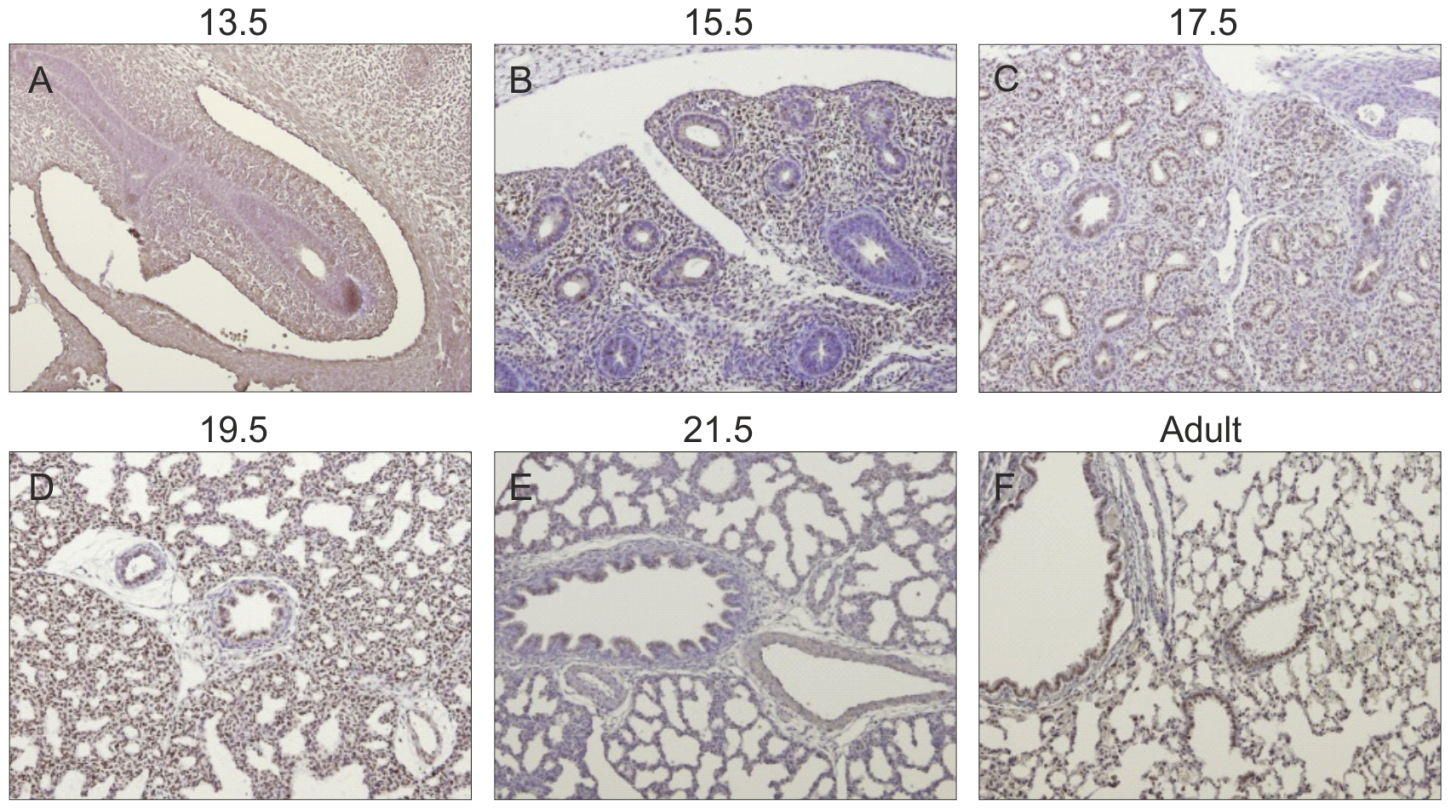
IL-11 expression pattern during fetal lung development. IL-11 is expressed throughout all stages of lung development studied, from early 13.5 dpc until late 21.5 dpc and also in the adult. Representative immunohistochemistry staining of (A–E) developing lung and (F) adult lung. Original magnification ×100.

**Figure 2:**
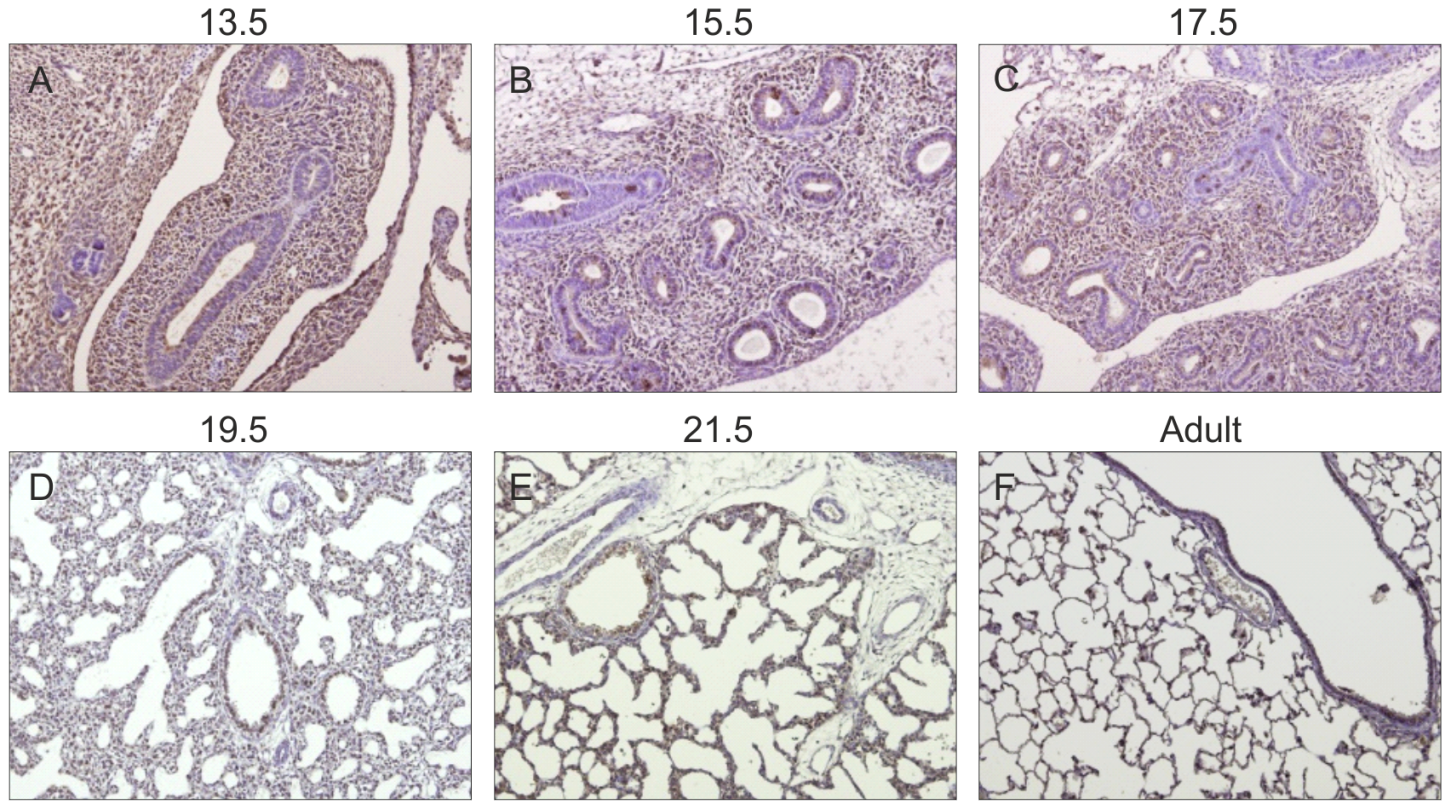
CLC expression pattern during fetal lung development. CLC is expressed throughout all stages of lung development studied, from early 13.5 dpc until late 21.5 dpc and also in the adult. Representative immunohistochemistry staining of (A–E) developing lung and (F) adult lung. Original magnification ×100.

**Figure 3:**
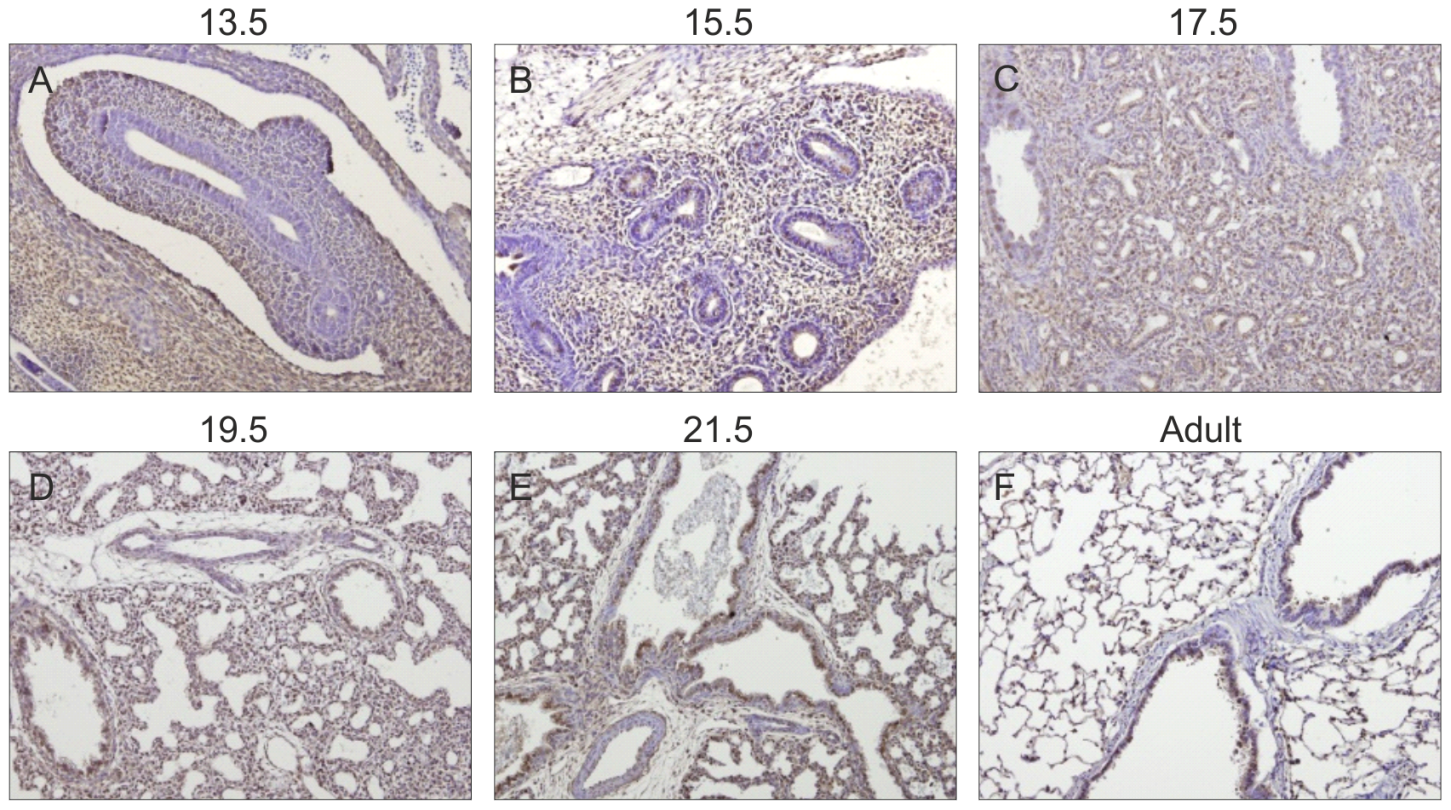
CNTF expression pattern during fetal lung development. CNTF is expressed throughout all stages of lung development studied, from early 13.5 dpc until late 21.5 dpc and also in the adult. Representative immunohistochemistry staining of (A–E) developing lung and (F) adult lung. Original magnification ×100.

**Figure 4:**
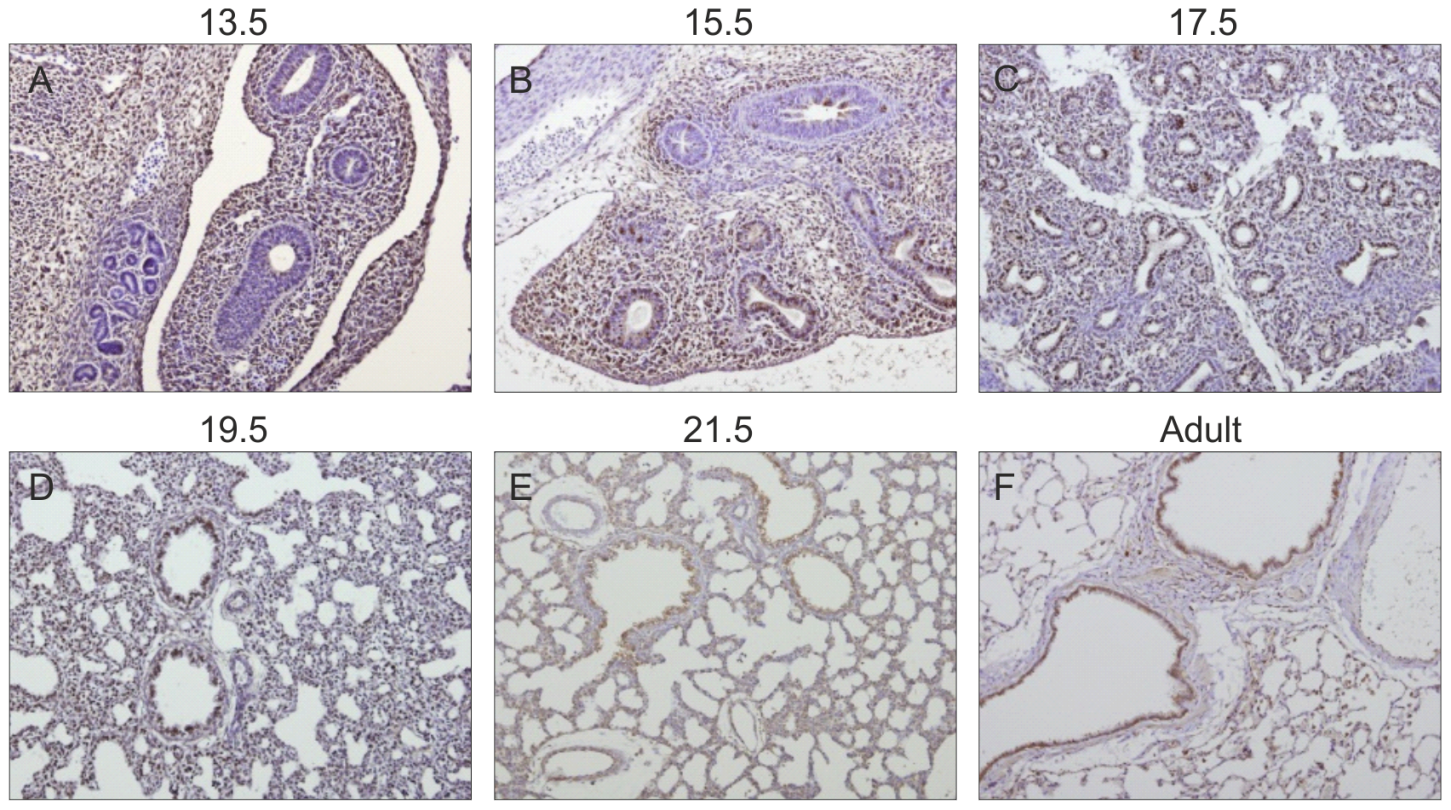
CT-1 expression pattern during fetal lung development. CT-1 is expressed throughout all stages of lung development studied, from early 13.5 dpc until late 21.5 dpc and also in the adult. Representative immunohistochemistry staining of (A–E) developing lung and (F) adult lung. Original magnification ×100.

**Figure 5:**
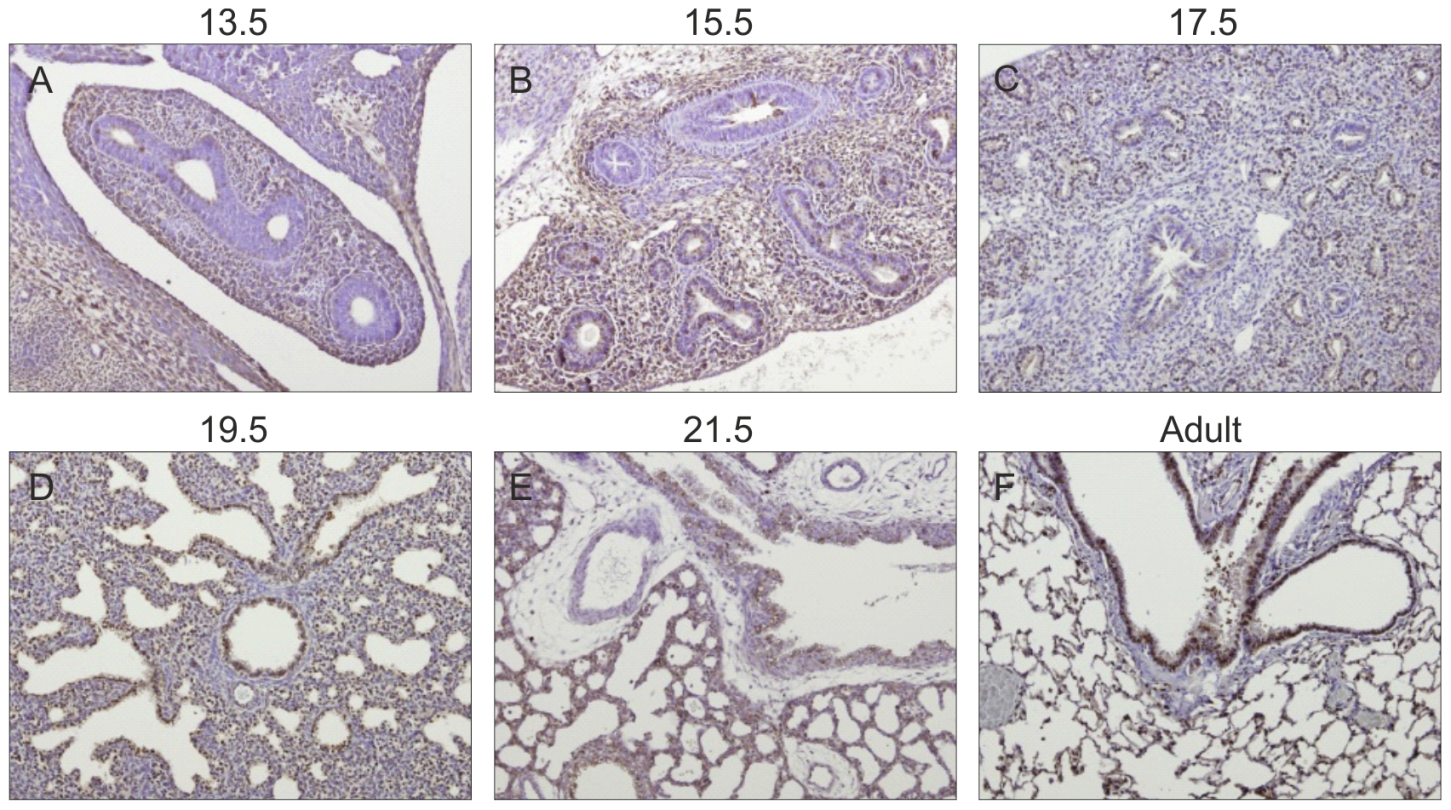
OSM expression pattern during fetal lung development. OSM is expressed throughout all stages of lung development studied, from early 13.5 dpc until late 21.5 dpc and also in the adult. Representative immunohistochemistry staining of (A–E) developing lung and (F) adult lung. Original magnification ×100.

**Figure 6:**
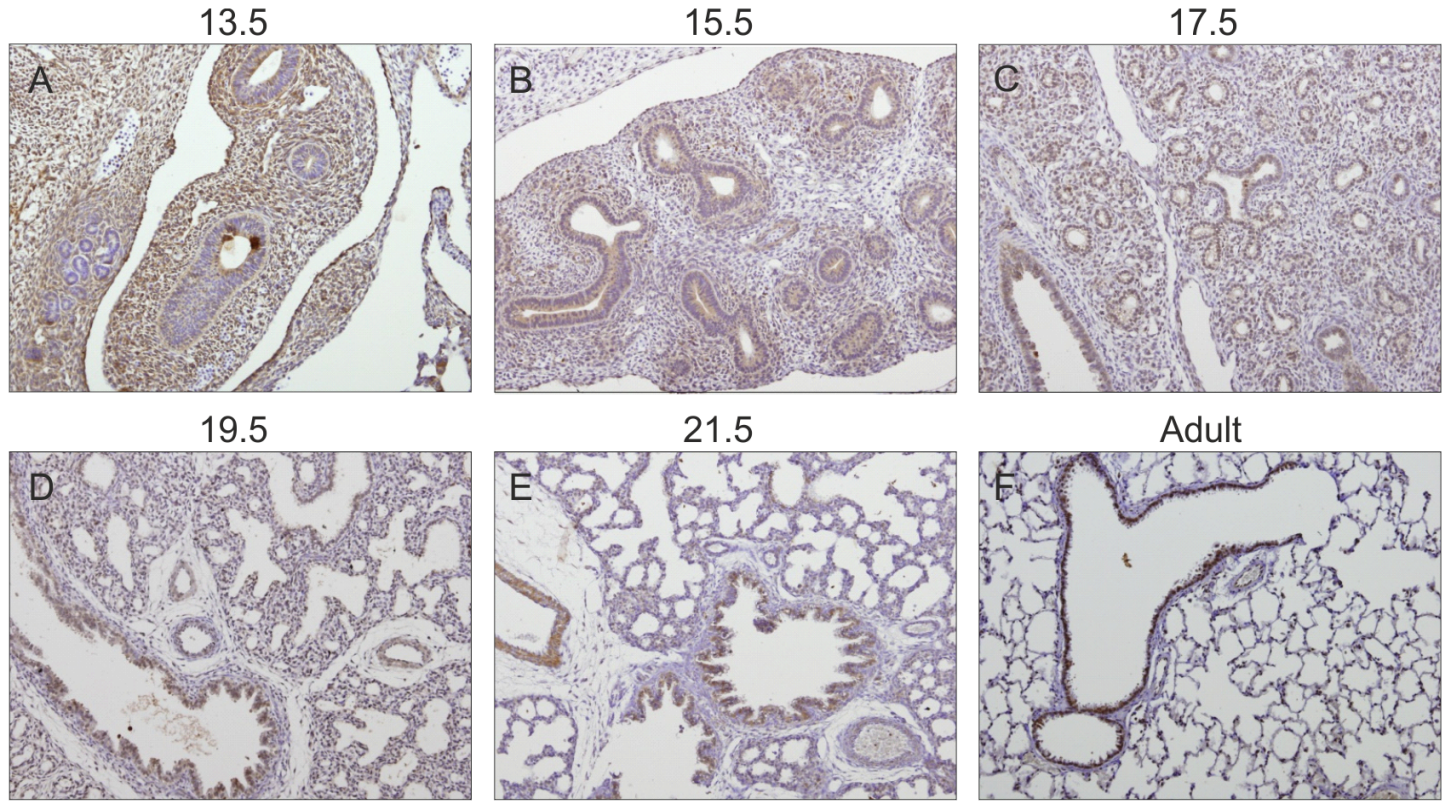
Gp130 receptor expression pattern during fetal lung development. Gp130 receptor is expressed throughout all stages of lung development studied, from early 13.5 dpc until late 21.5 dpc and also in the adult. Representative immunohistochemistry staining of (A–E) developing lung and (F) adult lung. Original magnification ×100.

Spatio-temporal protein expression pattern of IL-11, CLC, CNTF, CT-1, OSM and gp130 receptor were assessed in the developing lung at five gestational ages, specifically 13.5, 15.5, 17.5, 19.5 and 21.5 dpc and also in the adult lung tissue. Immunohistochemistry revealed that all these cytokines and their common gp130 receptor are expressed throughout all studied gestational ages in the fetal lung (Figures 1, 2, 3, 4, 5, 6). IL-11 is first mainly expressed in the undifferentiated mesenchyme at 13.5 and 15.5 dpc, however at late pseudoglandular stage, 17.5 dpc, immunostaining is observed in both mesenchyme and in the epithelial lining of proximal and distal airways. At late gestational stages, IL-11 is predominantly associated with both bronchial and alveolar epithelium, similarly to the adult tissue (Figure 1). CLC immunostaining is detected in the embryonic mesenchyme at 13.5 dpc, and remains predominant in this tissue at subsequent gestational stages; inversely as gestation progresses CLC epithelial expression is mostly observed, as illustrated at term as well as in the adult (Figure 2). CNTF expression is also detected as early as 13.5 dpc in the primitive mesenchymal tissue, from 17.5 dpc until term as airways develop CNTF epithelial expression gradually becomes predominant. In the adult, CNTF staining is restricted to the bronchi and alveoli (Figure 3). Mesenchymal CT-1 expression is present early in gestation, 13.5 dpc, and is concomitant to epithelial expression since 15.5 dpc onwards, throughout gestation CT-1 immunostaining in the airways is increasingly more apparent and also restricted to bronchi and alveoli epithelial lining in the adult (Figure 4). Similarly, OSM is also detectable since 13.5 dpc in the mesenchyme. At 15.5 dpc its expression is evident in both mesenchymal and epithelial embryonic tissues. OSM positive immunoreactivity in the epithelium of airways is clearly detected since 17.5 dpc and remains until term. Likewise, in the adult tissue protein expression is observed only in both bronchial and alveolar airways (Figure 5). Gp130 receptor protein is detected in the embryonic pulmonary mesenchyme at 13.5 dpc. Also early in development, at 15.5 dpc, epithelial expression of gp130 is already observed, and at subsequent gestational ages its expression remains predominantly associated with the developing epithelium. Additionally, gp130 immunostaining in the adult is also evident in the proximal and distal epithelial tissue (Figure 6).

### Role of gp130 cytokines on rat fetal lung development

**Figure 7:**
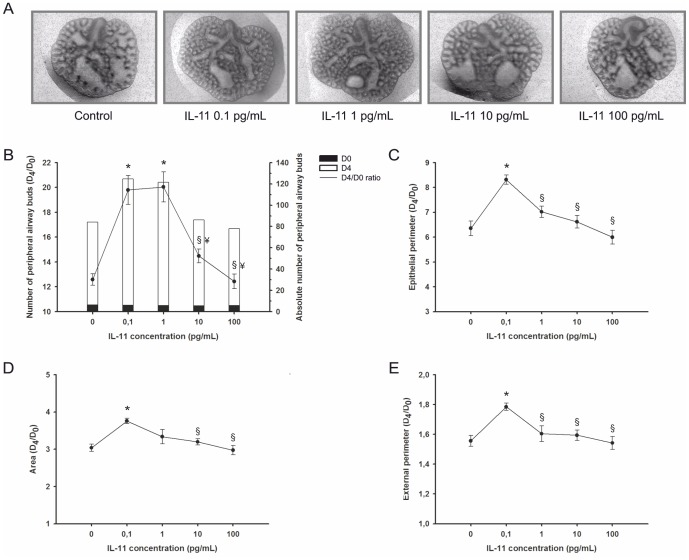
IL-11 supplementation studies in a fetal lung explant culture system. (A) Representative examples of fetal lung explants treated daily with increasing concentrations of recombinant IL-11, after 4 days in culture. Original magnification: ×25. (B) Number of peripheral airway buds ; (C) Epithelial perimeter; (D) Area; (E) External perimeter of lung explants treated with IL-11. Results are expressed as ratio of day 4 (D_4_) and day 0 (D_0_) of culture (D_4_/D_0_ ratio), and also as absolute number in D_0_ (black bar) and D_4_ (white bar). p<0.05: ^*^ *vs.* IL-11 at 0 pg/mL (control), ^§^ *vs.* IL-11 at 0.1 pg/mL, ^¥^ *vs*. IL-11 at 1 pg/mL.

This study aimed to clarify the role of gp130 dependent family of cytokines on lung morphogenesis. Thus, rat fetal lung explants cultured *in vitro* were treated daily with increasing concentrations of recombinant IL-11, CLC, CNTF, CT-1 and OSM. In Figure 7A, representative examples of fetal lung explants treated with increasing IL-11 concentrations, after 4 days in culture, are illustrated. IL-11 appears to have an enhancing effect on lung explant growth which is maximal at the lowest concentration tested, 0.1 pg/mL. In fact, an increase in the total number of peripheral airway buds (Figure 7B), epithelial perimeter (Figure 7C), area (Figure 7D) and external perimeter (Figure 7E) of lung explants was observed in all concentrations tested, except the highest, 100 pg/mL. In all the above mentioned morphometric parameters, explants treated with the highest dose presented the most similar effect to the control explants. In detail, increasing doses of IL-11 induced a biphasic effect in all the morphometric parameters assessed, generally the lowest dose of IL-11 enhanced explant growth, whereas increasingly high doses gradually reduced explant growth when compared to the maximal effect observed.

**Figure 8:**
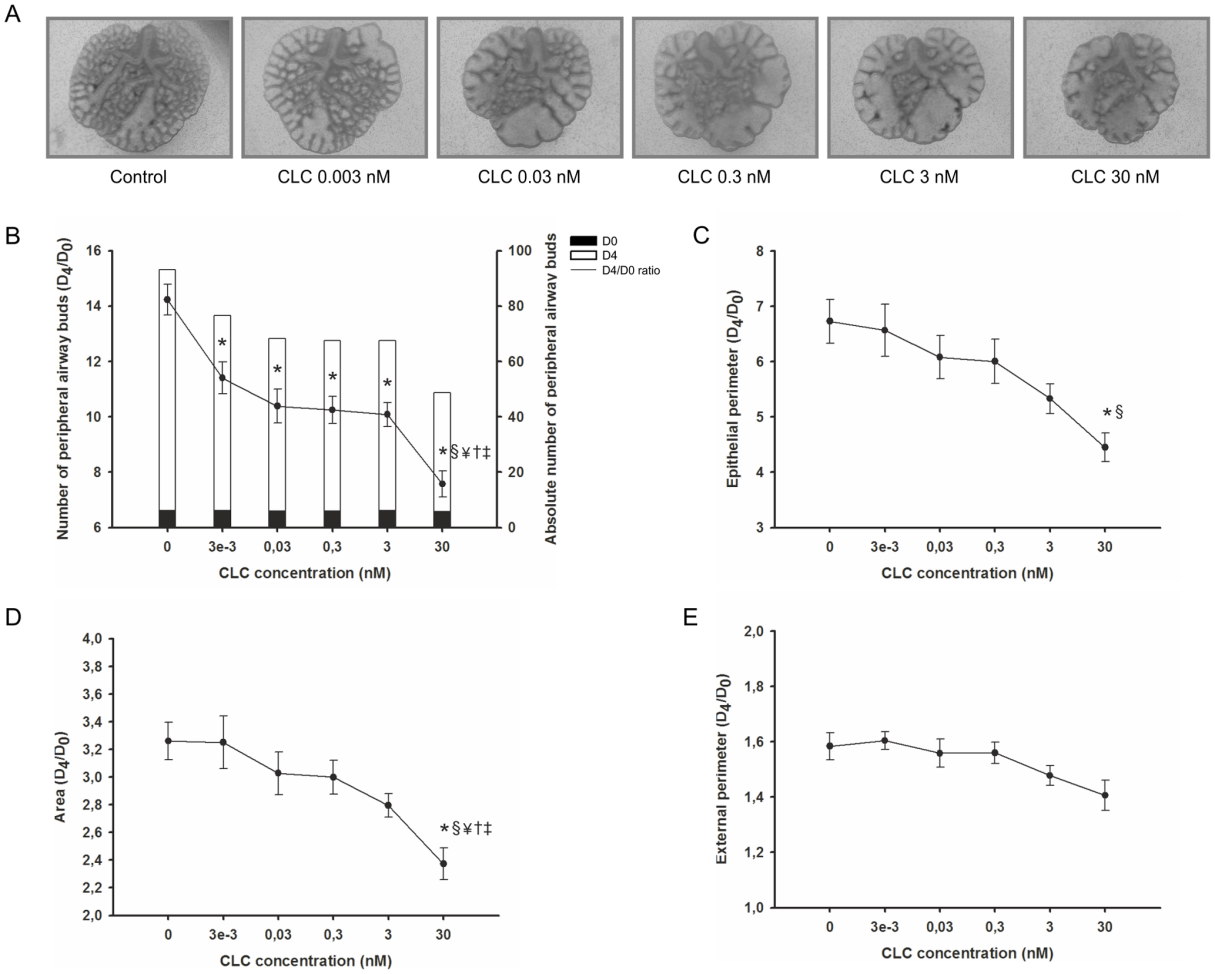
CLC supplementation studies in a fetal lung explant culture system. (A) Representative examples of fetal lung explants treated daily with increasing concentrations of recombinant CLC, after 4 days in culture. Original magnification: ×25. (B) Number of peripheral airway buds; (C) Epithelial perimeter; (D) Area; (E) External perimeter of lung explants treated with CLC. Results are expressed as ratio of day 4 (D_4_) and day 0 (D_0_) of culture (D_4_/D_0_ ratio), and also as absolute number in D_0_ (black bar) and D_4_ (white bar). p<0.05: ^*^ *vs.* CLC at 0 nM (control plus 4 mM HCl), ^§^ *vs.* CLC at 0.003 nM, ^¥^ *vs*. CLC at 0.03 nM, † *vs.* CLC at 0.3 nM, ‡ *vs.* CLC at 3 nM.

Regarding the role of CLC in fetal lung growth, in Figure 8A representative examples of lung explants treated with increasing CLC concentrations, after 4 days in culture, are illustrated. CLC appears to have a dose-effect inhibitory action on lung explant growth. In fact, a decrease in the total number of peripheral airway buds (Figure 8B), epithelial perimeter (Figure 8C), area (Figure 8D) and external perimeter (Figure 8E) of lung explants was observed in all concentrations tested, and this effect is most significant in the highest CLC concentration studied, 30 nM.

**Figure 9:**
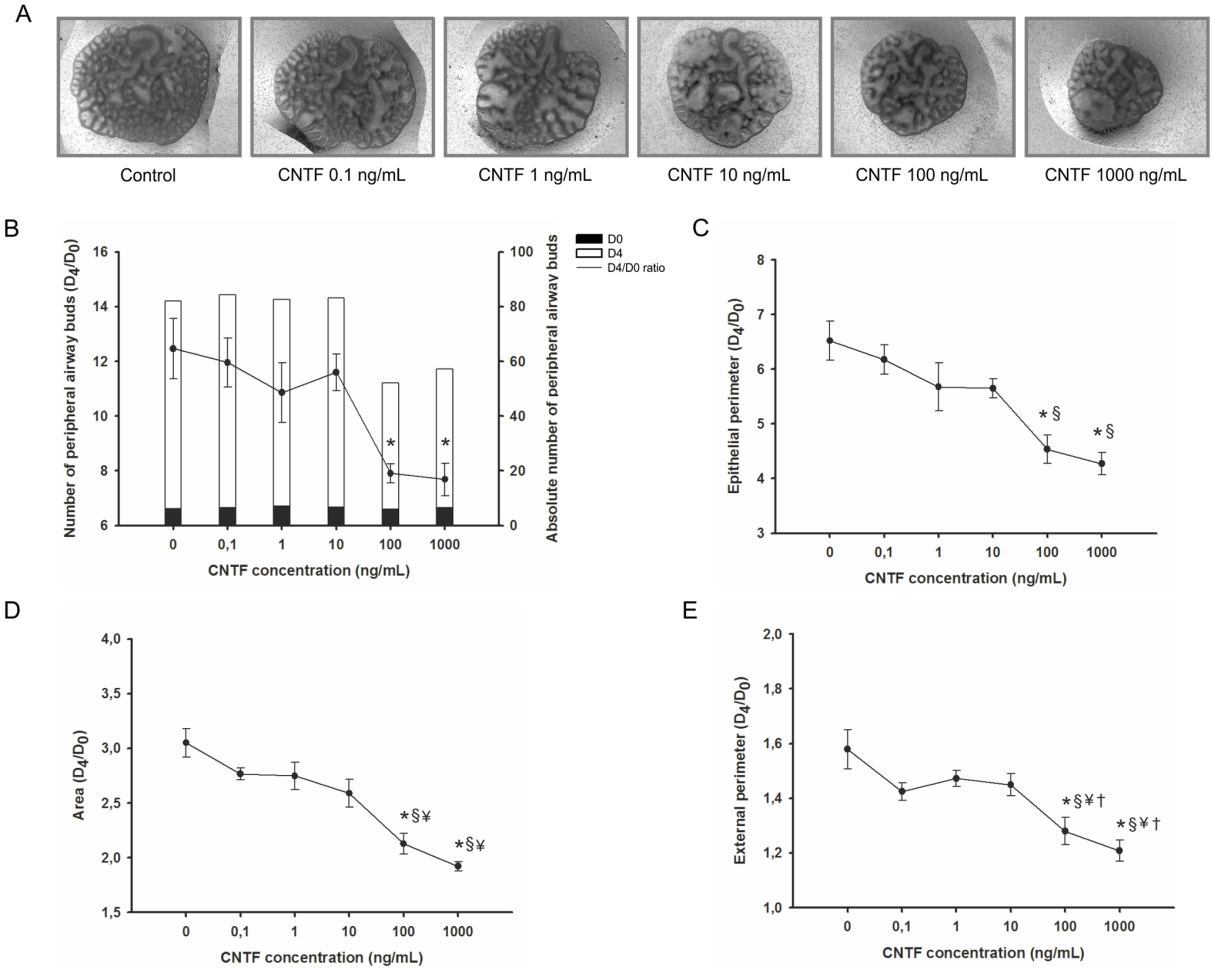
CNTF supplementation studies in a fetal lung explant culture system. (A) Representative examples of fetal lung explants treated daily with increasing concentrations of recombinant CNTF, after 4 days in culture. Original magnification: ×25. (B) Number of peripheral airway buds; (C) Epithelial perimeter; (D) Area; (E) External perimeter of lung explants treated with CNTF. Results are expressed as ratio of day 4 (D_4_) and day 0 (D_0_) of culture (D_4_/D_0_ ratio), and also as absolute number in D_0_ (black bar) and D_4_ (white bar). p<0.05: ^*^ *vs.* CNTF at 0 nM (control), ^§^ *vs.* CNTF at 0.1 ng/mL, ^¥^ *vs*. CNTF at 1 ng/mL, † *vs.* CNTF at 10 ng/mL.

In Figure 9A, representative examples of fetal lung explants treated with increasing CNTF concentrations, after 4 days in culture, are illustrated. CNTF appears to have an inhibitory action on lung explants growth, with the maximal effect induced by the highest CNTF concentration studied, 1000 ng/mL.

**Figure 10:**
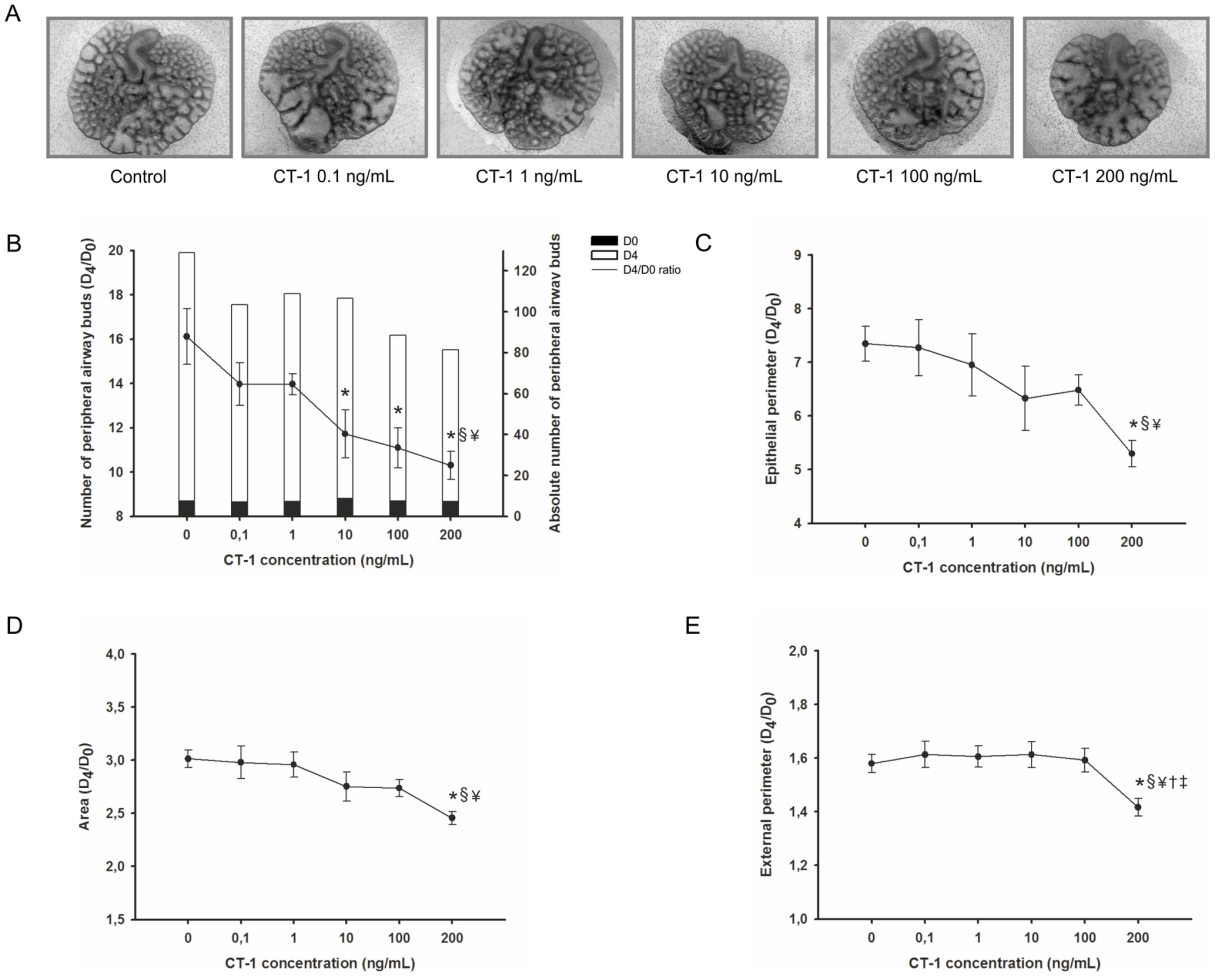
CT-1 supplementation studies in a fetal lung explant culture system. (A) Representative examples of fetal lung explants treated daily with increasing concentrations of recombinant CT-1, after 4 days in culture. Original magnification: ×25. (B) Number of peripheral airway buds; (C) Epithelial perimeter; (D) Area; (E) External perimeter of lung explants treated with CT-1. Results are expressed as ratio of day 4 (D_4_) and day 0 (D_0_) of culture (D_4_/D_0_ ratio), and also as absolute number in D_0_ (black bar) and D_4_ (white bar). p<0.05: ^*^ *vs.* CT-1 at 0 ng/mL (control plus 4 mM HCl), ^§^ *vs.* CT-1 at 0.1 ng/mL, ^¥^ *vs*. CT-1 at 1 ng/mL, † *vs.* CT-1 at 10 ng/mL, ‡ *vs.* CT-1 at 100 ng/mL.

Concerning CT-1, CT-1 appears to have a dose-effect inhibitory action on lung explants growth, as illustrated in figure 10A. In fact, a decrease in the total number of peripheral airway buds (Figure 10B), epithelial perimeter (Figure 10C), area (Figure 10D) and external perimeter (Figure 10E) of lung explants was observed in all concentrations tested, this effect is most significant in the highest CT-1 concentration studied, 200 ng/mL.

**Figure 11:**
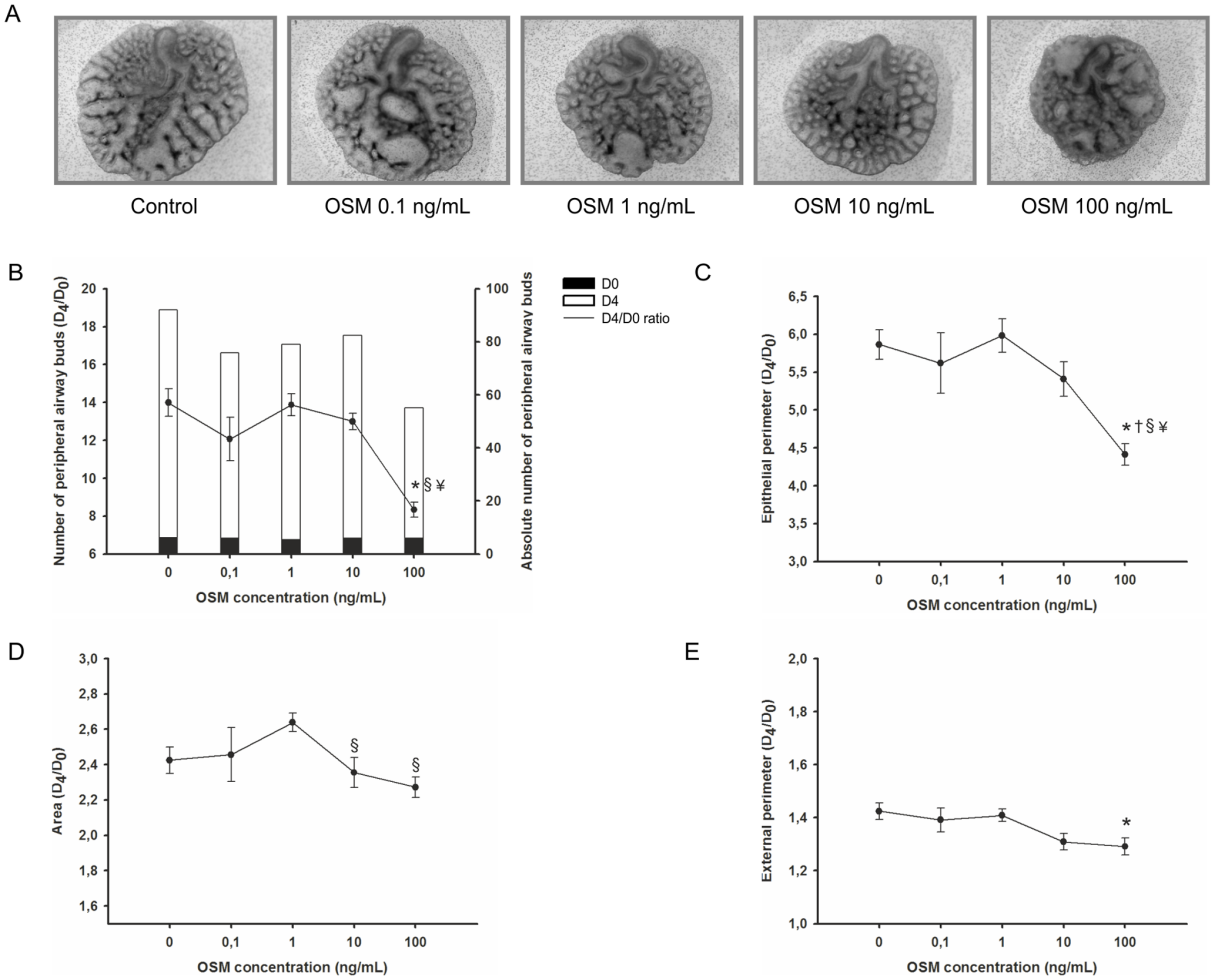
OSM supplementation studies in a fetal lung explant culture system. (A) Representative examples of fetal lung explants treated daily with increasing concentrations of recombinant OSM, after 4 days in culture. Original magnification: ×25. (B) Number of peripheral airway buds; (C) Epithelial perimeter; (D) Area; (E) External perimeter of lung explants treated with OSM. Results are expressed as ratio of day 4 (D_4_) and day 0 (D_0_) of culture (D_4_/D_0_ ratio), and also as absolute number in D_0_ (black bar) and D_4_ (white bar). p<0.05: ^*^ *vs.* OSM at 0 ng/mL (control), † *vs.* OSM at 0.1 ng/mL, ^§^ *vs.* OSM at 1 ng/mL, ^¥^ *vs*. OSM at 10 ng/mL.

As illustrated in figure 11A, OSM appears to have an inhibitory effect on lung branching. In fact, OSM at 100 ng/mL induces a significant decrease in the total number of peripheral airway buds (Figure 11B), epithelial perimeter (Figure 11C), area (Figure 11D) and external perimeter (Figure 11E) of lung explants.

### Gp130 cytokines supplementation effects on intracellular signaling pathways

**Figure 12:**
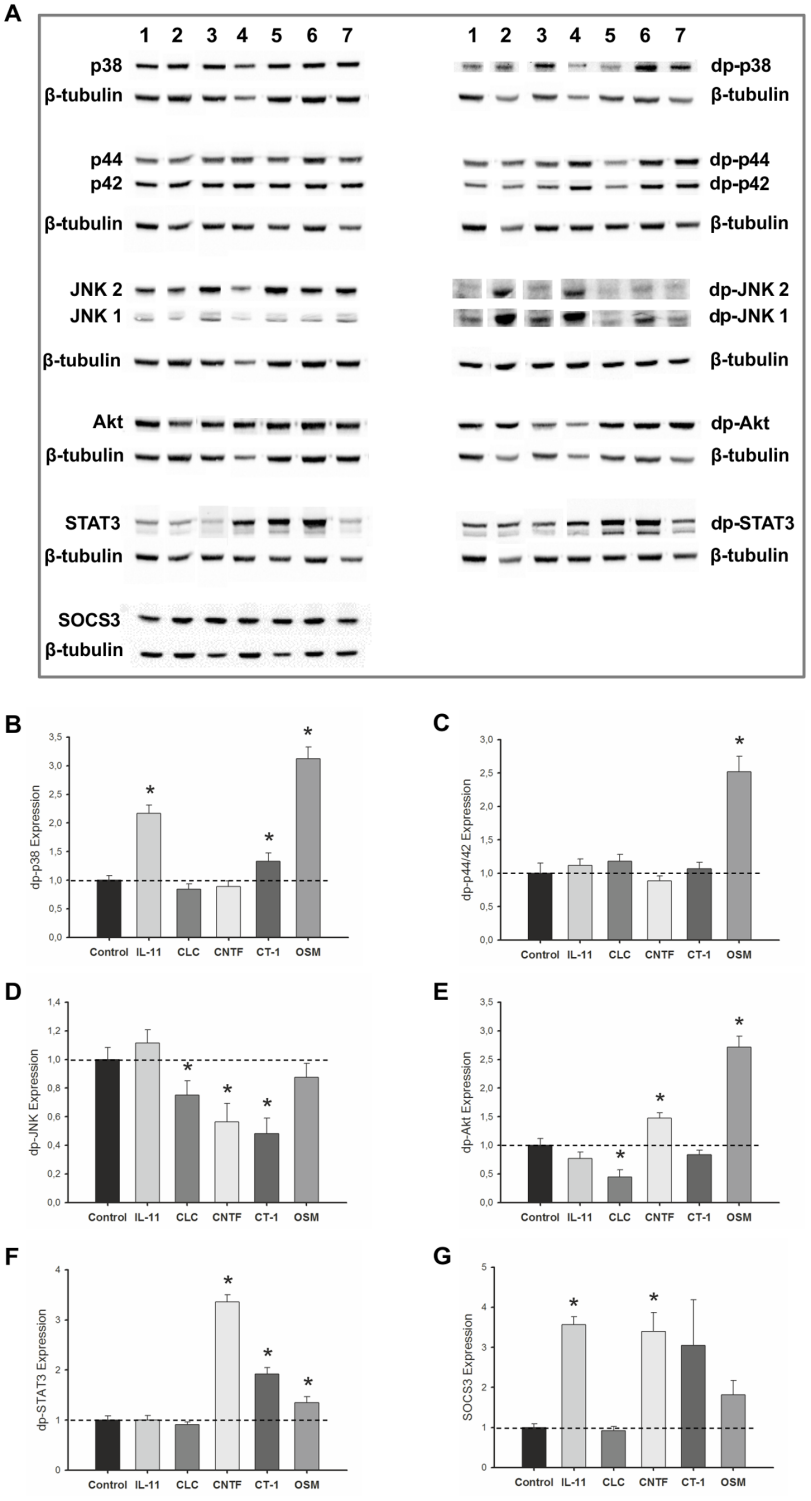
Analysis of intracellular signaling pathways that IL-11, CLC, CNTF, CT-1 and OSM supplementation mediates on lung growth. (A) Western blot analysis of p38, p44/42, JNK1/2, AKT, STAT3 and SOCS3, and also diphosphorylated forms of p38 (dp-p38), p44/42 (dp-p44/42), SAPK/JNK (dp-JNK1/2), AKT (dp-AKT) and STAT3 (dp-STAT3) in control (1), control plus 4 mM HCl for CLC and CT-1 lung explants (2) and treated with IL-11 at 0.1 pg/mL (3), CLC at 30 nM (4), CNTF at 1000 ng/mL (5), CT-1 at 200 ng/mL (6), and OSM at 100 ng/mL (7). Control loading was performed using β-tubulin (55 kDa). p38 corresponds to 38 kDa. p44/42 corresponds to 44 and 42 kDa, respectively. JNK1 and 2 corresponds to 46 and 54 kDa, respectively. AKT corresponds to 60 kDa. STAT3 corresponds to two bands, 79 and 86 kDa. SOCS3 corresponds to 30 kDa. Semi-quantitative analysis for (B) dp-p38, (C) dp-p44/42, (D) dp-JNK1/2, (E) dp-AKT, (F) dp-STAT3, and (G) SOCS3. Results are presented as arbitrary units normalized for β-tubulin and the respective control. p<0.05: ^*^ *vs.* control.

The receptors of gp130 cytokines directly control the activities of STAT, MAPK, and PI3K/AKT signaling pathways, and simultaneously activate SOCS negative feedback regulation. In order to further investigate gp130 cytokines effects on fetal rat lung growth, treated lung explants were evaluated for signaling modulation of these pathways. Pooled samples of lung explants individually treated with recombinant cytokines (selected due to its maximal effect either on stimulation or inhibition of explants growth) were used to assess protein expression levels of non-phosphorylated and phosphorylated forms of p38, p44/42 (ERK1/2), JNK, AKT and STAT3 by western blot, likewise SOCS3 protein expression levels were also assessed (Figure 12). Resulting signaling pathway alterations induced by gp130 cytokine stimulation are summarized in Table 1. On one hand, lung growth stimulation induced by IL-11 significantly increased p38 phosphorylation. On the other hand, inhibition of lung growth induced by CLC, significantly reduced JNK and AKT phosphorylation levels. Both CNTF and CT-1-induced inhibition of lung growth significantly stimulated STAT3 phosphorylation and decreased JNK phosphorylation. Additionally, CNTF treatment also induced a significant increase of AKT phosphorylation, whereas CT-1 treatment significantly increased p38 phosphorylation. OSM inhibitory effects on lung growth appear to be mediated by significantly increase of p38 and p44/42 phosphorylation, and also of AKT and STAT3 phosphorylation. Gp130 cytokines supplementation, comparatively to no supplementation, induced an increase in SOCS3 expression levels, except for CLC treatment. Both IL-11 and CNTF stimulation significantly increased SOCS3 expression.

**Table 1:** Gp130 cytokines effects on intracellular signaling pathways and lung growth.

Cytokine	Effect on intracellular signaling	Effect on fetal lung
IL11	↑ p38 phosphorylation; ↑ SOCS3 expression	Stimulation of lung growth
CLC	↓ JNK and AKT phosphorylation	Inhibition of lung growth
CNTF	↓ JNK phosphorylation; ↑AKT and STAT3 phosphorylation; ↑ SOCS3 expression	Inhibition of lung growth
CT-1	↓ JNK phosphorylation; ↑ p38 and STAT3 phosphorylation	Inhibition of lung growth
OSM	↑ p38, p44/42, AKT and STAT3 phosphorylation	Inhibition of lung growth

Analysis of the intracellular signaling pathways that IL-11, CLC, CNTF, CT-1 and OSM supplementation mediates on fetal lung growth. Note: (↑) (↓) arrows indicate increased and decreased, respectively.

### Gp130 cytokines supplementation effects on proliferation and apoptosis

**Figure 13:**
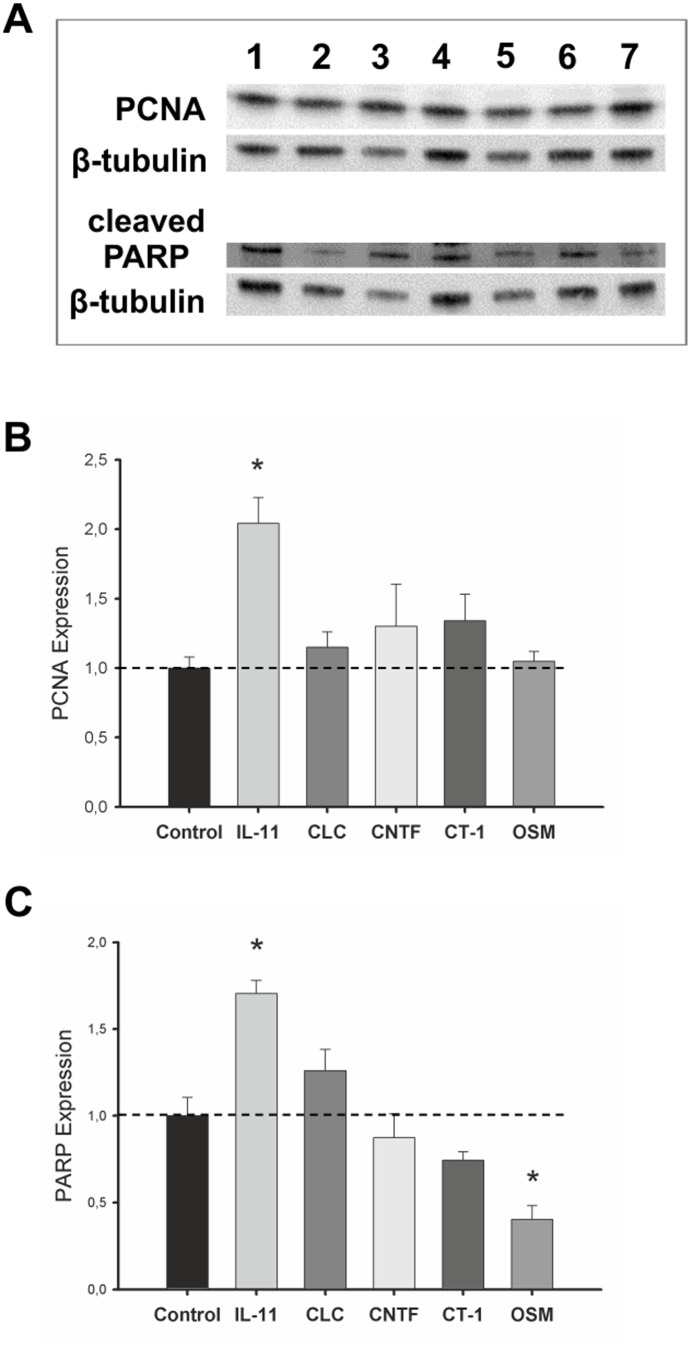
Effects of IL-11, CLC, CNTF, CT-1 and OSM supplementation on proliferation and apoptosis. (A) Western blot analysis of PCNA and cleaved PARP in control (1), control plus 4 mM HCl for CLC and CT-1 lung explants (2) and treated with IL-11 at 0.1 pg/mL (3), CLC at 30 nM (4), CNTF at 1000 ng/mL (5), CT-1 at 200 ng/mL (6), and OSM at 100 ng/mL (7). Control loading was performed using β-tubulin (55 kDa). PCNA corresponds to 36 kDa. Cleaved PARP corresponds to 89 kDa. Semi-quantitative analysis for (B) PCNA, p = 0.003, ^*^ *vs.* control and (C) cleaved PARP, p<0.05: ^*^ *vs.* control. Results are presented as arbitrary units normalized for β-tubulin and the respective control.

Gp130 cytokines stimulatory or inhibitory effects on fetal rat lung explant growth were further explored by assessing the protein expression levels of proliferation and apoptosis markers, PCNA and cleaved PARP respectively (Figure 13). Western blot was performed, using pooled samples of lung explants individually treated with recombinant cytokines. Concerning proliferation, IL-11 stimulatory effects on lung growth are concomitant with a significant increase of PCNA levels. Additionally, lung growth inhibitory cytokines, CLC, CNTF, CT-1 and OSM did not induce changes in PCNA levels relatively to no supplementation control (Figure 13B). In relation to apoptosis, inhibition of lung growth by CLC, CNTF, CT-1 and OSM induced a decrease in cleaved PARP, comparatively to growth stimulating-IL-11. Concurrently, IL-11 supplementation induced the highest expression level of cleaved PARP observed, and OSM the lowest, both significantly different relatively to control (Figure 13C).

## Discussion

The gp130 cytokine family collectively exhibits a broad range of physiological functions, including important roles in embryonic development. Recently, some of these cytokines, namely IL-6 and LIF, have been proposed to be mediators in fetal lung development [3]–[4], but otherwise little is known about the role of additional classical members of this family in the developing lung.

Moreover, information regarding protein expression patterns of these cytokines and their common gp130 receptor during embryonic rat pulmonary development is lacking from literature. Therefore, the present study demonstrated, for the first time, that gp130 receptor and its ligand cytokines are expressed during lung development. IL-11, CLC, CNTF, CT-1 and OSM are expressed at very early gestational stages of lung development, suggesting a biological role for these cytokines in early normal development of this branching organ. These cytokines share a similar protein expression pattern, in early stages of development with prevalence in undifferentiated tissues, all gp130 cytokines are expressed predominantly in the embryonic mesenchyme. Interestingly, as gestation progresses and airways develop and differentiate, gp130 cytokine expression becomes increasingly more restricted to both bronchial and alveolar epithelium. Concurring with our findings, several expression studies regarding gp130 cytokines, although referring mostly to IL-11, CLC, CNTF, CT-1 and OSM mRNA levels, have proved the expression of these cytokines in the murine adult lung [5], [18]–[24]. Furthermore our findings demonstrated that these cytokines also share a similar protein expression pattern in the rat fetal lung and, that for the most part of pulmonary development, gp130 cytokines expression is highly associated with both proximal and distal airways. This is found to be in agreement with further evidences that showed OSM [23] and CLC [25] expression in the pulmonary airway epithelium. It is also demonstrated that gp130 receptor protein is present in embryonic mesenchyme since early pulmonary development, and as gestation progresses its expression is predominantly associated with the developing epithelium, similarly to both the expression patterns observed for gp130 cytokines and LIFR in lung development [4].

In order to further clarify the role of the gp130 family of cytokines in lung branching morphogenesis, *in vitro* supplementation studies were performed individually. Thus, fetal lung explants were cultured with increasing concentrations of IL-11, CLC, CNTF, CT-1 or OSM, selected according to literature [26]–[35]. Supplementation studies showed that cytokines within the gp130 family can elicit opposite effects in lung explant growth. Such observation suggests that despite their shared use of the common receptor subunit gp130, these cytokines can generate contradictory signals in branching morphogenesis. Furthermore, intracellular signaling contribution to the effects of each cytokine on fetal lung growth were investigated by assessing non-phosphorylated and phosphorylated protein expression levels of several intracellular mediators, namely p38, p44/42, JNK, AKT, STAT3, and total SOCS3. It is well-established that numerous players account for the molecular basis of cytokine action, thus unsurprisingly in fetal lung development each cytokine proved to elicit the activation of either simple or combinatory signals from different signal-transducing pathways. Additionally, gp130 cytokines effects on lung explant growth were further explored by assessing proliferation and apoptosis levels.

In this study, it was demonstrated that IL-11 supplementation stimulates lung branching evidenced by increased number of peripheral airways buds, epithelial perimeter, area and external perimeter of fetal lung explants, whereas CLC, CNTF, CT-1 and OSM inhibit lung growth. Together with previously obtained results which demonstrated that IL-6 and LIF have opposite effects in branching morphogenesis, while the first stimulated lung explant growth the latter inhibited, the current study extensively contributes for a thorough comprehension of the role of these cytokines in the complex process of lung development.

Similarly to what was previously described for IL-6, IL-11 supplementation stimulated fetal lung branching. This stimulatory effect is concomitant with a significant increase in proliferation. IL11 supplementation also significantly, elicited the highest level of apoptosis observed. It is reasonable to expect an increase in proliferation in a branching stimulatory context mainly supported by epithelial development [1]. It is also known that apoptosis can be detected throughout the lung developmental stages, however it is most prominent during the pseudoglandular stage when branching morphogenesis occurs [36], [37], [38]. In addition, most cells undergoing apoptosis are reported to be located in the mesenchyme [36], [38], [39]. Therefore the combination of these processes is likely responsible for the observed IL-11 growth stimulation. Interestingly, IL-11 has been demonstrated to stimulate proliferation and differentiation of intestinal cells and to prevent apoptosis of epithelial cells [40], [41]. Regarding the lung, IL-11 is produced by a variety of structural cells (fibroblasts, epithelial cells, human airway smooth muscle cells] and eosinophils in response to a variety of stimuli [42], [43]. Indeed, IL-11 acts as a healing cytokine in the asthmatic airway and also provides protective effects against oxidant-mediated injury in fetal and adult lung [44], [45].

On the contrary, it was demonstrated that CLC, CNTF, CT-1 and OSM inhibit lung growth. Simultaneously, these inhibitory cytokines showed decreased apoptosis levels comparatively to stimulatory IL-11, moreover OSM presented a significant decrease in apoptosis relative to control. In agreement, others have previously reported diminished fetal lung branching in the presence of apoptosis inhibition [36], [46]. Several evidences point towards a role of these inflammatory cytokines in varied aspects of lung physiology. During development, CLC is expressed in lung, particularly in distal airway epithelium, as well as several other organs [47], suggesting important biological roles of this cytokine. In opposition to lung growth inhibition here demonstrated, during kidney development (also a branching organ), CLC promotes mesenchymal to epithelial conversion and nephrogenesis [28]. CNTF is described to be widely expressed in the adult [20], but during embryonic development, CNTF is specifically expressed in rat pineal gland and eyes [48]. This cytokine has been described to act as a lesion factor, preventing neuronal cell death and facilitating axonal regeneration after nerve injury [32]. CT-1 is expressed in both adult and fetal lung and also in numerous other embryonic and adult tissues [5], [21], [22], [49]. In opposition to the inhibitory effect on fetal lung growth described in this study, CT-1 has been related with hypertrophic and cytoprotective actions [49]. In fact, CT-1 has been related with chronic asthma, contributing to airway wall thickening and hypertrophy of airway smooth muscle [22]. Lastly, OSM is expressed in hematopoietic tissues, choroid plexus and limb during fetal life [50]. After birth, it is detectable in many other organs, and also the lung, specifically in alveolar and bronchiolar epithelium [23], [24]. Moreover, OSM is a potent mediator of lung inflammation and extracellular matrix accumulation [51].

**Figure 14:**
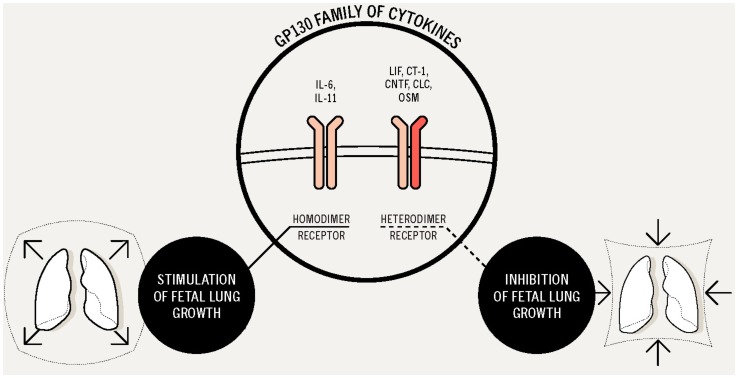
Overview of the role of gp130 family of cytokines in fetal lung development, cytokines signaling through gp130 homodimers (IL-6 and IL-11) stimulate fetal lung growth, whereas cytokines acting through a gp130 heterodimer receptor (LIF, CT-1, CNTF, CLC and OSM) inhibit lung growth.

Our findings on this dual contribution of gp130 family of cytokines, with inductive and suppressive actions in lung growth, clearly suggest a regulatory role in fetal lung development. Previously stressing a role for gp130 signaling during embryo development are many studies of transgenic and knockout mice for different components of this cytokine family or their receptors, which report defects in bone and neurologic development, disrupted placental architecture, hypoplastic development and a decrease in fetal liver hematopoiesis. The most severe phenotypes are displayed by mice lacking receptor components used by several members of the gp130 family. For example, gp130 or LIFR knockout mice die during development or shortly after birth [11], [52], [53]. Likewise, conditional gp130-mutant mice presented pulmonary defects and developed emphysema with increasing age [54]. In the present study, it was revealed that individual gp130-type cytokines can both enhance or inhibit fetal lung growth. Besides, providing specificity for individual cytokines in fetal lung development, these data underlines that cytokines operating through gp130 homodimers may induce different and even opposite biological responses than those operating through gp130 heterodimers. Both IL-6 and IL-11 receptors are gp130 homodimers and stimulate lung growth, whereas all the other receptors for this family of cytokines are gp130 heterodimers and inhibit lung growth (Figure 14). Thus, the main findings of this study stress the composition of these signaling receptor complexes as an important mechanism to acquire signaling specificity from pleiotropic-acting cytokines in lung development. This is in agreement with the well-documented fact that such cytokines with pleiotropic activities can also retain tissue-specific activities. In fact, several mechanisms can be accountable for generating and limiting those responses, specifically: cytokine restricted temporal and spatial release, differential expression of cell surface receptors and different signaling pattern between gp130 homodimers and heterodimers [55].

Cytokine signaling on a developing lung cell-specific context triggers diverging and non-overlapping intracellular signaling cascades. For instance, IL-11 stimulating effect on lung growth was associated with an increase in p38 phosphorylation. Interestingly, the stimulation of lung growth induced by IL-6 was also previously reported to be associated with increased p38 activation [4]. In the case of gp130 cytokines that exert an inhibitory effect in lung explant growth, diverse and combinatory intracellular signals are more frequent. Clearly this study emphasizes that also in lung branching morphogenesis, gp130 cytokine receptor activation is a rather complex means of initiation of signal transduction that leads to numerous possible signaling patterns able to elicit a similar biological outcome. CLC induced lung growth inhibition and concomitantly a decrease in the activation of JNK and AKT. Both CNTF and CT-1-induced lung growth inhibition is associated with activation of STAT3 and decreased JNK phosphorylation. CNTF additionally activates PI3K/AKT cascade whereas CT-1 activates p38. OSM inhibition of lung growth demonstrated to activate PI3K/AKT, different MAPK signaling pathways (p38 and p44/42) and also STAT3.

Unsurprisingly, gp130 cytokines fetal lung explant supplementation, comparatively to no supplementation, induced an increase in SOCS3 expression levels, except for CLC treatment. SOCS3 is rapidly induced following cytokine stimulation, both *in vitro* and *in vivo*. Additionally it is well-established that STAT1 and STAT3 contribute significantly to upregulate *socs3* gene [12]–[14], [56]. In agreement, we observe a significant increase in STAT3 activation concomitant with increased SOCS3 expression, comparatively to no stimulation, in CNTF, CT-1 and OSM treatments. Inversely, such is not observed regarding IL-11 and CLC stimulation, the first shows significantly increased SOCS3 expression and no apparent STAT3 activation, CLC stimulation did not elicit STAT3 activation neither SOCS3 overexpression. Considering that SOCS are induced via JAK/STAT pathway, which in its turn is initiated upstream by gp130 cytokines, and subsequently act preventing STAT phosphorylation, they ultimately suppress cytokine signaling in a classical feedback inhibition [13], [56]. Narrowing cytokine stimulation to a single dose, instead of a range when investigating alterations in STAT phosphorylation and SOCS expression, may be accountable for missing observation of the whole negative feedback loop response, and rather be restricted to observe partial cellular responses of this loop. This is likely to explain our diverse STAT3/SOCS3 signaling findings, because only a specific dose of a particular gp130 cytokine was analyzed. Moreover both, lung growth stimulating IL-11 dose and lung growth inhibiting CNTF dose significantly increased SOCS3 expression, proving that gp130 cytokines regulation mechanisms are induced independently of distinct physiological outcomes in fetal lung development.

The role of gp130 cytokine signaling negative regulation in the multiplicity of networks that are activated in response to these cytokines is often difficult to elucidate. Therefore we acknowledge, that future studies resourcing to other strategies such as gene expression profiling, blocking parts of the signaling cascade or using knockout technology in the context of fetal lung development would provide a more thorough insight on the gp130-induced signaling pathways and its regulation. Collectively, these results suggest that integration of the activities of multiple pathways might ultimately provide a balanced biological outcome intended to respond to a particular physiological situation.

In conclusion, in a similar way to IL-6, IL-11 acts in a gp130 homodimer receptor and it was demonstrated that stimulates lung branching. On the other hand, CLC, CNTF, CT-1 and OSM receptors are gp130 heterodimers and it was described that they inhibit lung growth. All these results demonstrated that cytokine signaling through gp130 homodimers stimulate, whereas cytokine signaling through gp130 heterodimers inhibit lung branching. This specificity of gp130-type cytokines might represent a regulatory mechanism of lung morphogenesis, intrinsic to this family of cytokines, in order to achieve the correct lung growth.
